# “If Not Me, Then Who?”: Exploring the Challenges Experienced by Front-Line Clinicians Screening for, and Communicating About, Domestic Violence in the Emergency Department

**DOI:** 10.1177/10778012231186816

**Published:** 2023-07-07

**Authors:** Sally Sargeant, Kathleen Baird, Amy Sweeny, Thomas Torpie

**Affiliations:** 1 Faculty of Health, Southern Cross University, Gold Coast, Australia; 2 School of Nursing and Midwifery, Griffith University, Gold Coast, Australia; 3 Emergency Department, Gold Coast Hospital and Health Service, Queensland, Australia

**Keywords:** domestic violence, screening, emergency department, patient communication

## Abstract

Screening for domestic violence in healthcare settings increases detection. The emergency department (ED) is one setting where victims frequently attend with injuries and illnesses sustained from violence. However, screening rates remain suboptimal. There is little research about how formal screening occurs, or how less structured interactions are negotiated within the ED context. This article explores this important, but non-mandatory procedure within the context of clinician–patient interactions in Australia. A descriptive qualitative study was undertaken with 21 clinicians across seven EDs in Australia. Thematic analysis was undertaken by two researchers. Results indicate a lack of confidence around DV screening, and tensions in clinicians initiating conversation while managing their own emotional stressors. No participants expressed knowledge of formal screening processes in their workplaces. Successful DV screening programs must provide clinicians with the tools to minimize perceived discomfort in initiating and sustaining conversations while accepting patient preferences regarding disclosure.

## Introduction

Domestic violence (DV) is increasingly recognized as a major cause of morbidity and mortality worldwide. Increased media reporting of major incidents and high-profile campaigns from the World Health Organization (2021) and Our Watch Australia (2021) have contributed to increased public awareness of violence against women. The COVID-19 pandemic brought DV into sharp focus, with concerns expressed for those enduring lockdowns with perpetrators (Galloway, 2020), and DV is also noted to be event-contingent, for example, alongside major sporting events (Livingstone, 2018). Health professionals in some jurisdictions are directed to DV management resources such as international guidelines (World Health Organization, 2013), national recommendations (e.g., National Institute for Health and Care Excellence, 2014) or state guidelines (e.g., Queensland Health, 2017). Such resources contain information relating to training staff in identifying and assisting with possible cases.

Opportunities exist—within acute and community-based healthcare—for staff to play critical roles to screen for and identify cases of DV. The purpose of screening in healthcare settings is to identify current or past experiences of violence so they can be referred to DV support services or be offered an intervention leading to beneficial outcomes (Nelson et al., 2012; O’Doherty et al., 2014). One health service environment in which DV can be identified is the hospital emergency department (ED), where victims are often assessed and treated (Murphy et al., 2016). However, there are many reported barriers to the identification and screening in broader areas of health service provision (O’Doherty et al., 2014) such as confidence in initiating conversations (Taylor et al., 2013), and lack of information about DV issues (Kirk & Bezzant, 2020). According to the World Health Organization and the National Institute for Health and Care Excellence, healthcare professionals should be aware and trained to identify, check for safety, provide support and refer those affected by DV (National Institute for Health and Care Excellence, 2014; World Health Organization, 2013).

An ED can be a busy, chaotic, hurried and highly stressful environment, and not immediately conducive to structured DV screening, but despite this patient interactions and clinical assessments may yield obvious “red flags.” These might include injuries being incommensurate with presenting history, or perpetrators refusing to leave a patient alone with a health professional. Research into DV screening in EDs varies considerably. It is hard to determine consensus on an optimal *modus operandi* due to many confounding factors that influence why screening may not occur. Certain comorbidities have unfortunately been noted to intersect and influence these deficiencies, such as patients who are diagnosed with psychiatric problems or substance use (Choo et al., 2010). Many policies, protocols and tools exist for DV screening, within and outside of an emergency care context; the Center for Disease Control (CDC) has mapped a considerable majority of these instruments (Basile et al., 2007). Some are clinician/nurse administered, others are self-report measures designed for patients to respond to statements about physical, sexual, and emotional violence. The number of items can range from short open questions to a longer series of specific ones. Some questionnaires are directed at specific populations (e.g., pregnancy, mothers of young children, male patients). Furthermore, there is considerable variation in the reliability and validity associated with screening instruments; the CDC document notes that the psychometric properties of many tools are unavailable.

An example screening tool is the partner violence screen (PVS), consisting of three brief questions, including “do you feel safe in your current relationship” and “is there a partner from a previous relationship that is making you feel unsafe now.” Vonkeman et al. (2017) investigated the likelihood of ED staff incorporating the PVS into their daily routine with all patients and how much they believed the tool would be beneficial in detecting DV. Although findings suggested that 46.3% of staff were “likely” or “very likely” to use the PVS, 29.1% were unsure with 7.24% and 3.6% respectively indicating that they were unlikely or highly unlikely to use it. Such a margin of uncertainty demands further interrogation as to why this might be the case and consideration of other information-gathering mechanisms. Other tools ask several questions but record dichotomous “yes” or “no” answers, which may not be useful if a victim does not recognize or believe they are affected despite health professionals’ suspicions.

Comparisons between face-to-face interviews and self-report instruments reveal fewer missing data when the former modality was used, but no other variables showed a distinction (Svavarsdottir, 2010). The brevity of screening tools (i.e., those that contain fewer items for response) could reasonably be assumed to be more effective, especially given environmental confounders within what may be a high-octane environment. However, this was demonstrated not to be the case in a comparison study of a single question and four-item Ongoing Violence Assessment Tool, in which the former did not significantly differ in positive screening results (Hoelle et al., 2014). The social action of screening itself was also aligned with a fear of making a patient's situation worse (such as anxiety about causing offense), however, research does not identify adverse events of screening (Houry et al., 2008).

Much of the literature about DV screening in EDs focuses on protocol development, reviews of notes and statistical analyses of case presentations. The impact of screening protocols has been shown to improve the identification of DV (O’Doherty et al., 2014) and many separate screening tools have since been developed. However, detailed examination of the factors behind protocol content and emergency physicians/health professionals’ engagement with screening is limited. Interview studies have addressed screening issues from systemic viewpoints with the aim of charting variations in health delivery models (Williams et al., 2017), or examining individual experiences of institutional processes (Ritchie et al., 2009). Further analysis of individuals’ attitudes, perceived abilities, and efficacy of DV screening in the ED is also limited. In examining the benefits of direct versus indirect questions, Ritchie et al. (2009) concluded that indirect approaches were more likely to foster patient trust leading to disclosure, but that screening should be universal and not confined to cases in which DV was suspected. This is slightly contradicted by a later study of indirect questions from a validated tool (SAFE-T) in comparison to direct questions regarding current abuse, which revealed that the former was likely more useful in eliminating instances of DV rather than detecting it, and concluded that indirect questioning would be less useful for patients in the early stages of an abusive relationship and who may not even realize that they are in that situation (Rhodes et al., 2007).

Such contrasts demonstrate the necessity to consider the utility of qualitative approaches in attending to sensitivities involved in DV screening. A statistical outcome relating to a screening tool does not always and deliver the nuanced meaning that may be unpacked when undertaking a more in-depth examination of health professional experiences of screening, whatever form that might assume. Other approaches have championed method triangulation as key to accessing information about institutional systems, personal circumstances, and phenomena associated with screening in an ED setting while sampling participants from multiple sites (Leppäkoski & Paavilainen, 2013), but caveats always surround the logistical difficulties in executing such research.

A solid corpus of qualitative research about DV screening in the ED understandably focuses on victims’ experiences of being screened, with most studies recommending that screening practices needed significant review due to patient disclosures being ignored, or evidence of victim-blaming (Gasser, 2008). While patents were satisfied with the treatment of their injuries, high levels of dissatisfaction persisted with how issues relating to abuse were managed in relation to staff confidence, competence, and resources. Other qualitative approaches are aligned with particular professions, with nursing and midwifery leading the inclusion of phenomenological inquiry into DV screening experience. An interview study with emergency registered nurses revealed a perceived lack of formal educational training in DV matters, and the motivations to screen were not always consistent or based on routine instruction from institutional hierarchy (Fay-Hillier, 2016). Midwives described feeling unprepared in undertaking screening (Eustace et al., 2016); although not always in an ED setting, participants indicated inconsistency of institutional/health service direction as a large barrier. A key enabler of screening was the continuity of care inherent in midwifery, which is absent from the acute emergency setting.

Detecting DV is one part of a clinical encounter, but the way in which it is documented is also contingent upon the success of mechanisms used to elicit information. When in environments that have the advantage of facilitating continuity of care, there is scope to document concern, inconsistent presentation, and recommendations for treatment and follow-up, potentially building a case that strengthens the evidence for incidences of DV and for considering possible outcomes. While the ED may be an environment in which symptom presentations may indicate DV, there may be limited opportunity to comprehensively record key information due to the aforementioned demands of an acute setting. This therefore raises additional questions as to how ED personnel raise concerns among each other, and how they record these concerns for future reference should this be necessary. At present there is minimal research about how screening or similar interactions are negotiated within the context of the ED. One possible avenue is to ascertain how ED personnel define DV to be able to act on suspicions. In response to the possibilities that ordinary presentations in the ED were assumed to be results of DV, attempts to explore the classification of physical assaults relating to IPV resulted in considerable variations in patient outcomes and consultation content (Olive, 2017). This research found that classifications of IPV were too high, and the other instances of significant injury were being missed, thereby negating the possibility of specialist interventions. Diagnostic thresholds may differ, but assumptions in recognition also reveal differences in psychological constructs associated with DV identification, a key example being the differences between survivors of IPV noting that fear impeded any intention to disclose, in contrast with ED nurses who were more likely to say that a victim was in “denial” (Watt et al., 2008)

The quality of interaction with other healthcare team members—within and outside of the ED—is noted to facilitate screening and management of DV, with cooperation and willingness to screen as a team rather than just as individuals being cited as a key enabler (Sweeny et al., 2023; Zijlstra et al., 2017). The notion of developing a “culture” of screening, however, was found to be fraught with tensions surrounding the expectations of high patient turnover at busy times, and ultimately recommend a protocol within that research setting.

Reviewing the literature reveals a somewhat cyclical conceptual pattern of results. On the one hand, protocol developments and evaluations provide insights into the structure questioning and effectiveness of particular questionnaires (whether validated or internally drafted checklists). Alternatively, more questions arise about the nature and experience of screening, at the individual and institutional levels, and while some of these questions have been answered to a degree, a notable result is often a recommendation for a protocol. Central to these questions is the interaction and cohesion between health professionals, which necessitates further experiential inquiry as to how this factor, along with other established barriers and facilitators of DV screening, can be optimally managed. Given the persistence of suboptimal DV screening in the ED (Fisher et al., 2020), this article aims to explore this important, but nonmandatory procedure within the context of interactions.

## Methods

A qualitative descriptive design was chosen as it provides a unique opportunity to gain deeper insight into communication encounters relating to conversations in the ED that extend beyond checklist development and implementation. Such a design was, therefore, considered appropriate to describe the experiences of clinicians asking patients about domestic and family violence.

### Setting

Seven government-funded EDs across metropolitan and semirural regions in one Australian state participated in the study. The settings varied including hospitals from North, Central, and Southeast of the state, tertiary trauma centers and teaching hospitals, community centers, and regional centers.

### Participants

Clinicians from seven ED sites were invited to participate in an interview. Inclusion criteria were that they worked in the last two weeks in a study ED and had worked at least 30 h per week in the clinical environment. Twenty-one clinicians agreed to participate and were contacted to confirm a date and time convenient for them to be interviewed.

### Data Collection

A semistructured interview was performed and included targeted questions about barriers to DV screening identified from a prior survey of clinicians (Sweeny et al., 2023). All interviews were conducted by the same researcher (an ED medical consultant) to ensure consistency, either in person or remotely to suit participants’ convenience. The interview guide included open-ended questions that were asked in a flexible sequence depending on the direction of participants’ responses.

A convenient date and time for an interview was arranged with participants and information about the research aims and interview questions were discussed. Verbal consent was obtained from each participant prior to the interview proceeding. Due to the distance and remote locations of participants, interviews were conducted by telephone apart from two local sites at which the interviewer attended. Each participant was interviewed once for around 30–60 min. The interviews were digitally recorded and transcribed verbatim.

### Data Analysis

A template analysis of the interview transcripts (King, 1998) was undertaken by one researcher (SS) to assess participants’ knowledge of DV screening procedures, and also to explore possible differences and similarities between professions and occupational settings.

Interview transcripts were also further analyzed by two researchers (KB and SS) using a process of inductive thematic analysis (Clarke & Braun, 2013). Each interview transcript was read several times to obtain familiarity with the data, using the techniques associated with the six-stage methods as outlined by Clarke and Braun (2013). Initial codes were independently generated within the transcripts and recorded as individual notes by the researchers. Coding across the transcripts was continued until all the data extracts were coded. Provisional themes were developed and refined as the data analysis continued over time (Clarke & Braun, 2013). The two researchers met to compare and discuss their findings, agreeing on the final themes.

### Ethics

Ethical approval was obtained from the appropriate state ethics committee (HREC/17/GCH/103). Governance approval was obtained at each individual site.

Consent to participate and permission to digitally record the interview was reconfirmed prior to the commencement of each interview. The anonymity of participants was assured, and a pseudonym was allocated.

### Funding

Funding to undertake this research was provided by the Emergency Medicine Foundation.

## Results

### Contextual Information

Throughout analysis and revisions to this article, it became clear that outlining the context of “screening” and domestic violence management was necessary. While this article refers to screening, it is essential to clarify its meaning in the context of these particular results. Screening for domestic violence can take a structured form using validated questionnaires that are used in several clinical environments and may be adapted accordingly. However, in this study, when participants were asked about their experiences of screening, they were unaware of any formal DV screening processes within the institution in which they were based. This does not suggest that there were no broader procedures/policies in place, but it is important to note as the subsequent experiences presented here are based on personal recollections of unstructured conversations that were not framed by a formal screening process.

Twenty-one clinicians (seven doctors, 14 nurses) from seven sites were interviewed. Their clinical experience varied from 6 months to >30 years. Most of the participants had postgraduate qualifications, and all were currently working in the ED. A more detailed list of participant characteristics is presented in Table 1.

**Table 1.** **.** Participant Demographics.

	**Role**	**Years qualified**	**Age**	**Sex**	**Qualifications**
**1**	Student nurse	n/a	27	F	Undergraduate bachelor's degree
**2**	Clinical nurse	11 years	34	F	Undergraduate bachelor's degree
**3**	Registered nurse/part-time lecturer	9 years	30	F	Undergraduate bachelor's degree/postgraduate degree
**4**	Nurse practitioner	20 years	40	F	Undergraduate bachelor and postgraduate master's degree
**5**	Emergency registrar	>7 years	29	F	Medical training
**6**	Clinical nurse consultant	10 years	33	F	Postgraduate certificate and master's degree
**7**	Acting clinical nurse	8 years	29	F	Undergraduate bachelor's degree
**8**	Senior registrar	9 years	34	F	Undergraduate bachelor's degree (nursing) and postgraduate medical degree
**9**	Nurse practitioner	20 years	43	M	Overseas qualification—unspecified
**10**	Nurse practitioner	12 years	45	F	Undergraduate bachelor and postgraduate master's degree
**11**	Clinical facilitator (nurse education)	20 years	41	F	Postgraduate qualification
**12**	Registered nurse	>30 years	50	F	Predegree hospital-based training
**13**	Registered nurse	19 years	41	F	Undergraduate bachelor's degree and postgraduate speciality training
**14**	Medical consultant	14 years	39	M	Medical bachelor and speciality training
**15**	Registered nurse	30 years	52	F	Predegree hospital-based training
**16**	Medical (senior house officer)	6 years	32	F	Undergraduate bachelor medical degree
**17**	Medical registrar	7 years	29	M	Undergraduate bachelor's medical degree
**18**	Nurse practitioner	10 years	37	M	Undergraduate bachelor's and postgraduate master's degree
**19**	Medical consultant	11 years	35	M	Undergraduate bachelor's medical degree
**20**	Medical consultant	17 years	42	M	Undergraduate bachelor's medical degree
**21**	Registered nurse	>30 years	52	F	Predegree hospital-based training

The seven EDs were in hospital sites that were administered within one state jurisdiction in Australia. All seven hospitals were within the public healthcare system. Of the seven, three were large metropolitan teaching hospitals, and the remaining four served rural areas. Table 2 presents information relating to geographical location and approximate ED presentations in a 12-month period (2021–2022). The Australian classification of rural and/or remote geographic areas is specifically important to note here. Generally, any area outside of major cities is classified as rural, but this is further divided into terms “outer regional,” “inner regional,” “remote Australia,” and “very remote Australia.” The four hospital sites designated as rural were all classed as “outer regional,” however one was still situated in a city with a population nearing 200,000. While this may not equate to a perceived definition of rurality outside of Australia, it is appropriate to note as the geographical area covered by a designated “rural” service is larger than that of a metropolitan area.

**Table 2.** Characteristics of Hospital Sites.

	Setting	ED presentations in a 12-month period	Teaching hospital
1	Metropolitan	∼35,700	Y
2	Metropolitan	∼15,300	Y
3	Metropolitan	∼18,000	Y
4	Rural (outer regional)	∼3400	N
5	Rural (outer regional)	∼11,000	N
6	Rural (outer regional)	∼10,500	N
7	Rural (outer regional)	∼12,500	Y

### Thematic Analysis

Interviews with participants led to four themes. Three of these themes focus on the active components of interpersonal communications that the participants recalled relating to their direct experiences of and hypothetical assumptions of conversations relating to DV. However, the first theme—*Individual and institutional knowledge acquisition*—provides further context about participants’ knowledge and inexperience of training relating to DV screening practices. From a deductive analytic standpoint, the theme demonstrates the informational contexts relating to the sites where participants worked, and also individual recollections relating to learning about screening, from past experience to current occupational situations.

#### Theme 1: Individual and Institutional Knowledge Acquisition

All of the participants indicated that they were not aware of DV screening practices in their current ED workplace. Furthermore, most of them indicated that they had received no training, either specifically in DV screening as a distinct practice, or in more general communications relating to DV management/detection. If any training was mentioned, the narratives contained a conspicuous absence of certainty about the circumstances relating to the content or organization of training. An example from a newly qualified nurse states:

Outside of […] undergrad and in my post-grad the—we touched on it, to do with kind more psychology and social work and ethics, but beyond that, no, I would say. (#4 Nurse)

While this quotation was from someone recently qualified, other recollections demonstrated a similar uncertainty, with the following response from another nurse with over 10 years’ experience:

We’ve had an in-service, I think, once, a long time ago, done by the social workers, but other than that, it is just sort of on the job*.* (#10 Nurse Consultant)

In contrast to the nursing examples, a mid-career medical registrar recalled having some training that was specific to another clinical specialty (Obstetrics and Gynecology) and was able to situate the training in relation to knowledge required for specialist clinics:

I had some training as an ONG intern as part of an introduction to the antenatal clinic, and that was about it. (#17 Medical Registrar)

The mode of delivery of any recalled training was mentioned by some staff with medical backgrounds, but these indicated that online training was preferred.

I think we did some online stuff, you know, as part of … mandatory training, but if we actually had face-to-face training or something, no. We did not have anything like that. (#5 Emergency registrar)

One of the common phrases that occurred with reference to training and knowledge acquisition was “on the job,” implying a less formal training context and acquiring knowledge from peers and specific cases:

It was more just informal in our department, and it was more designed on how to ask the person at the point of triage, how to get them away from their partner, how to pick up signs of if the partner's there talking on their behalf, and it was all to do with getting an opportunity to question them about their safety away from the partner. (#21 Registered Nurse)

Such descriptions of “informal” knowledge acquisition relating to DV appeared across the responses, and there were no differences between those who worked in metropolitan or rural contacts. There were also no obvious differences in descriptions of training experience (or lack thereof) relating to how many years’ experience individuals had in their respective professions. Participants who were medical rather than nursing staff showed more, albeit limited, awareness of DV screening and recalled instances of training, but their accounts did not demonstrate certainty.

Having presented some contextual basis of participants’ knowledge of and training experiences relating to DV within the ED, the analysis now turns to a more detailed examination of mostly recalled and occasionally hypothesized interactions. The following themes 2, 3, and 4 are presented from an inductive analysis as described in the method section.

#### Theme 2: If Not Me Then Who?

This theme addresses who should potentially screen, or initiate conversations relating to DV with the patient, and how such communications are operationalized in the ED setting. Interviewees mostly defaulted to a named profession, rather than a particular individual:

I guess that is more the social workers’ job, from my point of view, it is not really something that I would feel comfortable and confident doing. I would not even know where to start. (#2 Clinical Nurse)

I just really think it should be the nurses …. I think that they probably have, generally, the best sort of relationships, I guess, with the patients because they are there with them, but most doctors—some doctors can be really blunt and not very good at that kind of stuff, and I just do not think that, maybe—you know, especially if they are women, they may not want to talk to that sort of stuff about a male—to another man. (#1 Student Nurse)

The gendered assumptions in the latter quote provoke further consideration of the role of gender stereotyping in clinical communication, but an implicit bias is likely present towards victims of DV being mostly female and the ease of communication being spearheaded also by a female.

When not deferring to professional clinical groups as being ideally suited to screening, other participants directly hypothetically positioned themselves as initiating conversations relating to DV:

I would gently try and talk to them about it, preferably on their own without the potential perpetrator or anybody else with them and basically, just try and explore their history around their injuries or their presentation and offer both the likes of social work service, if they do admit to it, or just leave an open conversational point regarding whether they would want to talk to me further. (#14 Consultant)

In this hypothetical context a structure of perceived conversation is proposed with the inclusion of offering access to specific services. However, another account detailing an actual verbal exchange in similar circumstances demonstrates a different and less straightforward outcome:

And this lady had come in, and her story did not add up to her injuries. And she made a comment, and I commented something back, and I cannot remember what it was now. But she then said to me ‘You think my husband has done this to me don’t you?’ and I just said ‘I'm not making any judgments. You're in a safe place, so if you wish to talk’ and then eventually she did come out and say that her husband had bashed her up. And it was just because her injuries did not match the story she had first told. (#15 Registered Nurse)

Through the reported speech this account exemplifies the tensions that may emerge when trying to broach the subject. Conveying suspicions of DV results in defensive response, which may not always lead to full disclosure. This leads to the issue of conversational unease in exploring potential DV situations, and what might circumvent this.

#### Theme 3: Conversational (Un)Ease

In response to interview questions about what would facilitate screening in terms of initial conversations, participants referred to a broad range of possibilities that focused on individual interactions through to institutional influences. Some offered suggestions about the nature of questioning:

Simple questions are always good because generally people do not get upset or offended by them, and if we say we ask them to all people then it is part of just our normal assessment tool, I do not think people would be offended by it, as long as we let them know that, that we are not just targeting a certain group of people maybe—although some people are just—they might be that fragile that they do not want to answer questions anyway. (#12 Registered Nurse)

Acknowledging simplicity is a key element but this account refers to a routine assessment tool that is universally administered, thereby promoting conversational ease with the implication that such conversations form part of usual practice. By contrast, other accounts revealed that divided attention and feelings of helplessness contributed to perceived ineffective screening:

I was helpless and largely giving treatment and opening the door to say, ‘Did anything happen? How did this happen?’ Saying, ‘Is there anything going on at home? We are always here to talk.’ But unfortunately, they were not open to discussion about anything. And beyond that, I suppose, I screened lightly and suspected and unfortunately had a lack of engagement from the patient. (#16 Senior House Officer)

The attempt to initiate a structured screening process (or inquiring about the possibility of DV) results in a self-categorized determination of screening “lightly” and differs from the previous extract of implied protocol/procedure. Although the respondent describes a lack of conversational reciprocity from the patient, the description suggests an attempt to simultaneously screen for DV while administering treatment, which inevitably affects conversational ease and screening efficacy. It also may suggest that some clinicians may lack the confidence and/or experience to delve deeper into conversation with a patient who is unwilling to disclose a history of DV at the first time of asking.

Some of the participants discussed the added difficulty of asking patients about DV when English was not their first language and the need to engage a third person in what is a very private conversation:

The interpreter service is great; they do a really good job, but I find it really difficult to engage with my patient through an interpreter, and I think it is also difficult to be able to build a rapport with your patient when you do have a third party in the room with you; it is just a little awkward. (#5 Registered Nurse)

Others considered the discursive strategies that enabled a desired effect from third parties, such as attempting to speak to patients alone:

I do remember being more junior and feeling either awkward, or worried about how to bring it up, or not sure how to ask the partner to leave, in a nice way. And I think it is about picking up those sort of phrases to say, hey you want to go and grab a cup of coffee, and we will just finish talking, or something like that. And ways to get the person to leave. Those kinds of easy ways out that you pick up through seeing other people doing it. So I think I feel okay about it. Still not great, or still not confident, because I have not done it that much. (#20 Consultant)

Many considerations emerge from this account with the underlying uncertainty of competence and/or incompetence. The rhetorical devices described to speak with the patient alone were observed by colleagues. Despite these suggested ways to make a difficult conversation less so, confidence remains low. The mention of feeling junior within the context also raises questions that do not simply align with competence, but that also hint at communications with other more senior health professionals in the ED setting.

The conversational un/ease described in these data extracts, and the implied uncertainly of the efficacy of their actions leads to another phenomenon described in screening interactions—deciding the point at which to cease anything relating to a DV screening or related conversation.

#### Theme 4: Letting Go and Managing Nondisclosure

As participants described what some of their screening attempts “looked like,” they also related instances of nondisclosure and how this manifested in the ED:

It is a fine line between being the advocate for the patient but also knowing that there is other things involved at the time. And because we obviously want what is best for them and to keep them safe but it is, yeah, it is very difficult if they would not admit or accept what is happening and, yeah, I find personally it is a little bit frustrating*.* (#7 Acting Clinical Nurse)

The multifactorial influences upon failure to obtain disclosure clearly pose emotional and practical difficulties.

While some ED staff acknowledged the right of patient to choose not to disclose understanding that for some, they were just too frightened to be honest about how they sustained their injuries:

that is their right to not disclose—if they do not want to disclose it. Yeah, if they are scared and they do not particularly want to or change anything. But I do think that if we have a high suspicion that this is happening like we said before that we should be able to contact somebody, just to potentially touch base with the patient once they are discharged and just check-in … but we do have a duty of care … (#2 Clinical Nurse)

The reference to “making wins” showcases a fractured sense of success relating to the screening process, whatever form that process may take. However, it also implies that a particular mindset is needed to alleviate inner feelings and outward expositions of frustration. Participants derived a sense of “incompleteness,” not only in conducting screening or related conversation, but also with reference to their own emotional responses in managing what is perceived to be an unsuccessful interaction:

You can only give the information that you have and the person's willingness to pick that up or not is 100% their decision. Obviously, we want the best for the patient, but ultimately you do your best and make wins when you can make wins, but yeah, I think, it is probably a good mentality to have within the circumstances, because if you show frustration over their circumstances, I think it is probably less likely to disclose things in the future. (#18 Nurse Practitioner)

While acknowledging the internal conflicts about whether to discontinue screening due to lack of disclosure, other participants situated the sense of incompleteness as a possible consequence of institutional congestion (or lack thereof):

So I think if you do not have someone step in and follow that up and make sure that they get the help that they need, to follow through on whatever plan was made, I think probably does form a big part of not completing that cycle. (#15 Senior House Officer)

It is unclear who the “someone” refers to in the extract—an individual or a representation of an external agency. However, the account also raises the topic of various cycles of completion needed to achieve effective DV screening in the ED context. Even if an ED health professional successfully screens a patient and obtains disclosure, a disconcerting aspect remains in letting go and hoping other agencies may follow up.

## Discussion

The common themes relating to conversations about DV that emerged from the 21 interviews were all associated with confidence about conversations with patients, and managing their own emotional stressors associated with the topic.

It is evident from the participants’ interview accounts that dissonance exists between the intention and actual reporting of suspected DV. In this sample, only one participant discussed the use of any policies or procedures which might guide their screening endeavors. The absence of clear protocols (inter and intrainstitutional) and a lack of training and education contribute to the uncertainty described by individuals suspecting DV from a patient narrative that lacks coherence or that is elicited in circumstances that are not conducive to open disclosure.

Clinical communication protocols are common within clinical practice. Handovers, for example, present a clear example of communicating necessary information (situation, background, assessment, and recommendation). Similarly, structured forms of communication exist to have difficult conversations, such as breaking bad news or conducting motivational interviewing. In broader contexts, campaigns exist to allow individuals to communicate to others that they feel threatened or vulnerable, for example, “Ask for Angela” (Australian Government, 2021) in the specific context of bartenders who can arrange exit strategies. It is, therefore, surprising that such a communication structure is not overt within emergency rooms or wider clinical contexts. The need for a rhetorical structure applies to two contexts—eliciting information from patients and communicating with fellow health professionals depending on the situation.

The nuances of conversation relating to DV management in the ED clearly need further investigation but given the lack of confidence and certainty revealed in the interview data here it is abundantly clear that institutional rhetoric and procedures need to be developed and promoted effectively. This needs to occur through appropriate and regular training, rather than depending on residual memories of formal university degree courses in which anything relating to DV/IPV training is a small feature within a complex operational landscape (Valpied et al., 2017). Regardless of the complexity of the environment, prevalence rates of domestic violence in EDs remain higher than those found in most other healthcare settings (Sprague et al., 2014). A study from the United States found women experiencing DV are 4 times more likely to present to an ED than nonabused women (Kothari et al., 2015), thereby, reinforcing the importance of and the value of screening program in this environment.

Few Australian studies have reported on established programs for screening presentations in the ED. A recent evaluation study conducted in three EDs in New South Wales over a 6-month period, found there were 12,131 presentations to the ED by 9,177 eligible women (Spangaro et al., 2020). Nineteen percent had two or more presentations to the ED during the study period. The rate was higher in rural areas at 24%. Of the 1,119 forms completed for analysis, 251 (22.4%) forms indicated that screening had not been conducted for a variety of reasons, including the presence of a partner or family member. Forty-five forms indicated that the patient had declined to answer the screening questions. This study had an overall screening rate of 11.4%, indicating that there nearly 90% of patients entering the three EDs were not screened for DV. There were differences found in the screening rates, with the Metropolitan sites having higher rates of screening, with the most likely explanation of the difference being the social worker presence located within the ED.

Spangaro et al. attempted to incorporate DV screening in EDs by introducing a simple tool to screen all adults, or all women at a minimum. However, only 11.4% of women were screened, indicating the complexity of this intervention. In Australia and the United States, the tool of choice at this time is the HITS tool (National Institute for Healthcare and Excellence, 2014), also used by Spangaro et al. (2020). A better administration method for screening could be either computerized or face-to-face. From a consumer standpoint, the preferred administration method of screening appears to be computerized from one review and meta-analysis representing six studies across various healthcare settings and countries (Japan, Canada, and the United States), although detection rates did not significantly vary by type of administration (Hussain et al., 2015).

The present study has limitations, most of which reside in the variability of the participant sample of different levels of professional experience and roles. A qualitative paradigm naturally embraces subjectivity, but nonetheless emphasizes the shared conversational difficulties between different groups of staff. Further work can, and should, continue to explore the experiential nature of communication in the context of DV screening, to enable nuances and specific inclusions, both in screening tool development and administration. Additionally, the variations in participant characteristics and hospital settings in this study indicate that further work on exploring differences and similarities between such variables is of value—especially in what would appear to be an absence of formal training relating to screening.

## Conclusion and Recommendations

Findings from this study and previous research would indicate that history taking and clinical examinations for DV in the ED remain insufficient. The burden of disease related to DV presentations is large enough to warrant screening in the ED especially as the ED can be the first point of entry for victims. A high proportion of women presenting to the ED will have one or more comorbidities with mental health being the most common (Zakrison et al., 2017). While the debate continues as to whether the ED is a suitable environment for routine screening for DV, it is clear that a need exists to promote the development and the use of structured protocols to facilitate open conversations. The purpose of this is twofold. First, protocol promotion and clearly communicated implementation are imperative to maximize the efficacy of screening, to attain information central to the appropriate care for and subsequent management of DV victims. Staff training should not be an isolated event; it is necessary to repeat relevant training over the course of clinicians’ practice, rather the restrict it to the confines of sessions within degree programs (as identified in our participants’ accounts). Secondly, overt training, guidelines and protocol development are also essential to minimizing clinicians’ perceived discomfort in initiating and sustaining difficult conversations. The research highlighted two unmet needs. These included the promotion and embedding of structured DV protocols in their clinical settings (the use of formal, validated processes), and second, training on initiating and sustaining difficult conversations that fall outside of formal screening. A successful DV screening program must provide clinicians with the tools to minimize clinicians perceived discomfort in initiating and sustaining difficult conversations, and advocate acceptance that it is the patient's preference to disclose or not disclose.
